# Telomere shortening during human septic shock: influence of sepsis mediators, role in organ failures, and septic myocardial dysfunction

**DOI:** 10.1186/s13054-021-03818-9

**Published:** 2021-11-18

**Authors:** Keyvan Razazi, Elisabeth Marcos, Sophie Hüe, Laurent Boyer, Serge Adnot, Armand Mekontso Dessap

**Affiliations:** 1 Service de Médecine Intensive Réanimation, Hôpitaux Universitaires Henri Mondor, AP-HP, 94010 Créteil, France; 2 GRC CARMAS, Faculté de Santé de Créteil, IMRB, Université Paris Est Créteil, 94010 Créteil, France; 3 Département de Physiologie, DHU ATVB, Hôpitaux Universitaires Henri Mondor, AP-HP, Créteil, France; 4 U955, INSERM, Université Paris Est Créteil, 94010 Créteil, France; 5 Service d’Immunologie, Hôpitaux Universitaires Henri Mondor, AP-HP, 94010 Créteil, France

Leucocyte telomere length (LTL) is widely considered a marker of cellular ageing. Telomere attrition has been involved in cardiovascular disorders as a result of inflammatory stress [1], but has been scarcely evaluated in acute settings. Septic shock is associated with an overwhelming inflammatory reaction that may be involved in the genesis of organ failure, including septic myocardial dysfunction [2]. The present study aimed to assess whether septic shock is associated with telomere attrition and evaluate the role of sepsis mediators and the impact on organ failures. Fifty-five patients free of chronic heart failure who met septic shock criteria (as defined according to the ACCP/SCCM Consensus Conference) were prospectively included at the medical intensive care unit of Henri-Mondor University Hospital (Créteil, France).

LTL was measured by QuantStudio™ 6 Flex Real-Time quantitative PCR System (Applied Biosystems, Foster, CA); see Table 1 legend. LTL was assessed in septic shock patients (in 55 and 24 patients on day-1 and day-2, respectively) and in 55 healthy controls matched to septic shock patients for age (± 3 years) and gender.

**Table 1:** Characteristics of patients with septic shock according to leucocyte telomere length at day 1 (*n* = 55)

	Lower LTL (*n* = 27)	Higher LTL (*n* = 28)	*P* value
*Clinical characteristics and comorbidities*			
Age (years)	64 [53–74]	64 [49–72]	0.60
Male gender, *n* (%)	20 (74%)	12 (43%)	0.02
Chronic obstructive pulmonary disease	0 (%)	1 (4%)	> 0.99
Chronic kidney disease requiring long-term dialysis	2 (7%)	2 (7%)	0.97
Liver cirrhosis	4 (15%)	3 (11%)	> 0.99
Mc Cabe and Jackson class			0.49
0	11 (39%)	12 (43%)	
1	10 (37%)	13 (46%)	
2	6 (21%)	3 (11%)	
SAPS II at ICU admission	50 [41–79]	59 [39–79]	0.83
Community acquired infection	13 (48%)	16 (57%)	0.50
Lung source of infection	14 (52%)	13 (46%)	0.69
Bacteraemia	11 (41%)	13 (46%)	0.67
Surgery	6 (27%)	4 (16%)	0.48
*Organ failures*			
Sequential organ failure assessment score	10 [8–13]	11 [8–13]	0.83
Arterial lactates (mmol/L)	2.9 [1.5–5.5]	2.9 [1.4–4.9]	0.87
PaO_2_/FiO_2_ ratio (mmHg)	164 [115–350]	188 [109–260]	0.79
Serum creatinine (mmol/L)	201 [84–338]	164 [84–264]	0.35
Septic myocardial dysfunction	10 (37%)	9 (32%)	0.70
*Outcome*			
Mechanical ventilation	25 (93%)	24 (86%)	0.67
Dialysis for acute renal failure	6 (22%)	4 (14%)	0.50
ARDS	18 (67%)	13 (46%)	0.18
ICU acquired infection	12 (43%)	9 (32%)	0.41
Death in ICU	15 (55%)	13 (46%)	0.49
Death in hospital	15 (55%)	16 (46%)	> 0.99

The telomere repeat copy number to single-gene copy number (T/S) ratio was determined using the comparative Ct method (T/S = 2^−ΔΔCt^) with 36B4 gene for normalization (acidic ribosomal phosphoprotein PO, a single-copy gene). Data are number (percentage) or median [first quartile-third quartile]

*LTL* leucocyte telomere length, *SAPS* simplified acute physiology score, *PaO*_*2*_ partial pressure of oxygen in arterial blood, *FiO*_*2*_ fraction of inspired oxygen, *ARDS* acute respiratory distress syndrome, *ICU* intensive care unit

Septic myocardial dysfunction (SMD) was defined as a depressed left ventricle ejection fraction (< 45% or when an inotrope infusion was needed to achieve a value ≥ 45%) on echocardiography performed on day-1 or day-2 of septic shock [2].

We assessed plasma concentration of 24 putative sepsis mediators on day-1, including inflammatory markers (IL-1α, IL-1β, IL1-RA, IL-6, IL-10, IL-12, IL-15, IL-17, IL-33, IFN-γ, TNF-α, CD40L, HSP70, sFAS, sFAS ligand, sST2, granzyme, TRAIL, PAI1, and VEGF), chemokines (IL-8, MCP1), and adhesion molecules (sVCAM, sICAM). sST2 and the remaining sepsis mediators were measured with human magnetic Luminex screening assay (R&D, Bio-Techne, Lille, France), and a multi-analyte Milliplex human cytokine kit (Millipore Corporation, Billerica, MA, USA), respectively, and were analyzed using fluorescence intensities [2].

Organ failures and patient severity were assessed using the Sequential Organ Failure Assessment (SOFA) and Simplified Acute Physiology Score II (SAPS II) score, respectively. Spearman bivariate correlations were used to build a focused principal component analysis (FPCA; “psy” package in R), using LTL as the dependent variable and allowing a simple graphical display of correlation structures.

LTL was similar between controls and septic shock patients (Fig. 1a). We observed a correlation between LTL and age in the control group as expected (Spearman’s rho = − 0.29, *p* = 0.04) but not in the septic shock group (rho = − 0.03, *p* = 0.82). Table 1 shows the clinical characteristics of septic shock patients according to LTL (below or above median value) on day-1; all variables were similar between groups, except for more female gender in patients with higher LTL. There was no statistically significant correlation between LTL on the one hand and SMD (Fig. 1b), SOFA score, SAPS II score, or sepsis mediators (except for sST2, sFASL and granzyme) on the other hand (Fig. 1c). LTL was similar in septic shock survivors and nonsurvivors at day-1, but decreased between day-1 and day-2 in survivors (Fig. 1d).

**Fig. 1:**
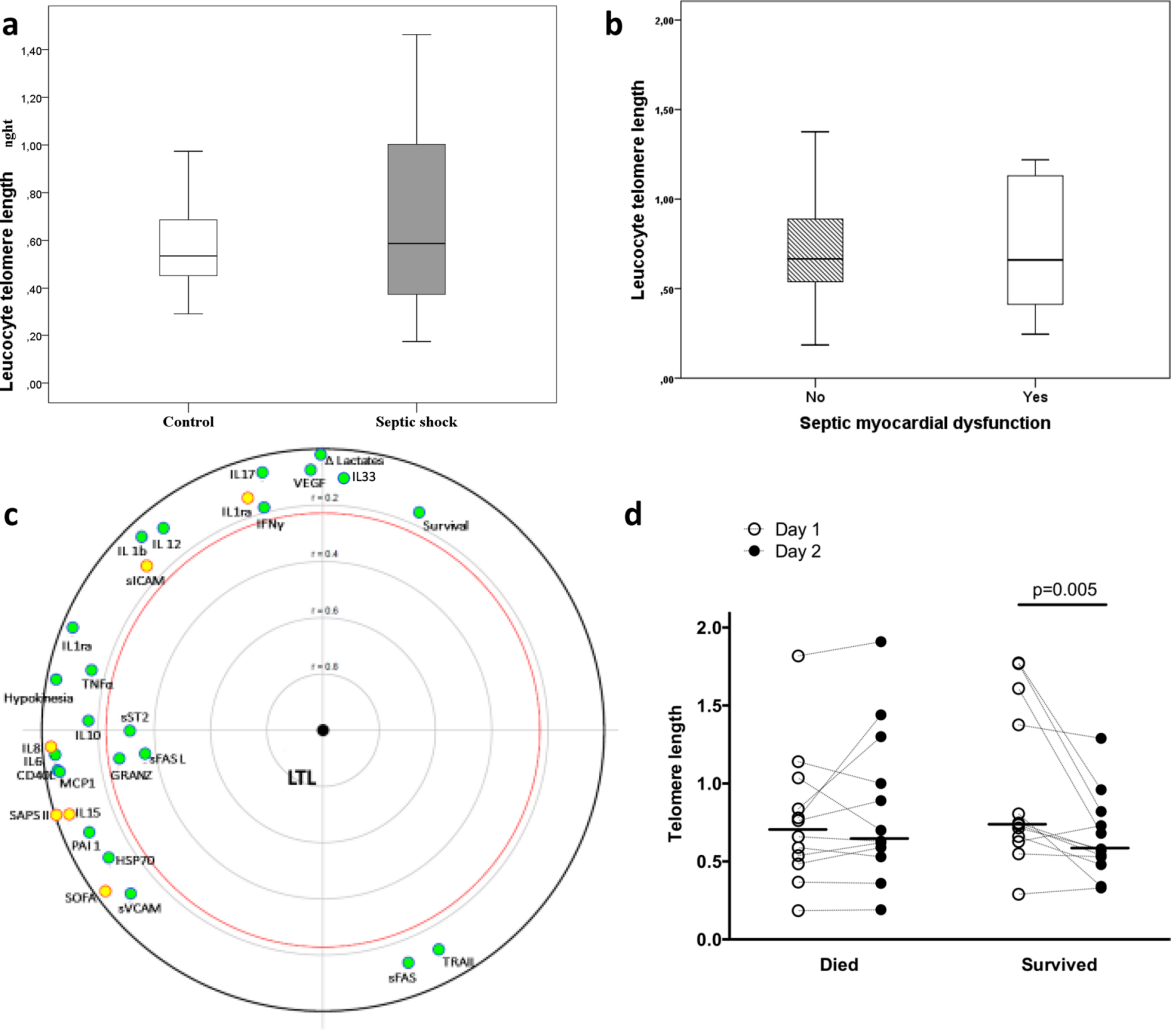
Leucocyte telomere length in controls and in patients with septic shock (**a**); LTL in septic shock patients with or without septic myocardial dysfunction (**b**); Focused principal component analysis (FCPA) for the association between leucocyte telomere length on the one hand, and sepsis mediators, Sequential Organ Failure Assessment (SOFA), Simplified Acute Physiology Score II (SAPS II), lactate clearance (Δ lactate) and survival on the other hand (**c**); LTL at day-1 and day-2 in patients with septic shock according to intensive care unit survival (**d**). FPCA is a simple graphical display of correlation structures focusing on a particular dependent variable. The display reflects primarily the correlations between the dependent variable and all other variables (covariates) and secondarily the correlations among the covariates. The dependent variable (LTL) is at the center of the diagram, and the distance of this point to a covariate faithfully represents their pairwise Spearman correlation coefficient (using ranked values of continuous variables). Green covariates are positively correlated with the dependent variable. Covariates significantly correlated with the dependent variable (with a *p* value < 0.05) are inside the red circle. The diagram also shows relationships between covariates as follows: correlated covariates are close (for positive correlations) or diametrically opposite vis-à-vis the origin (for negative correlations), whereas independent covariates make a right angle with the origin

LTL during septic shock may be determined by factors other than the inflammatory mediators we herein assessed; a prominent role for oxidative stress needs to be assessed in future studies. The association of LTL with FasL is in accordance with a previous study showing a decreased production of FasL after TCR/CD3 signaling of senescent T cells [3]. Senescence was also associated with reduced expression of the effector molecules granzyme and perforin [4]. The decrease in LTL in septic shock survivors may be explained by a relative hyperfunction of leucocyte against infection in this subgroup [5]. We did not find a correlation between LTL and organ failures, as previously reported by Liu et al. [6].

## Data Availability

The datasets supporting the conclusions are included within the article.
